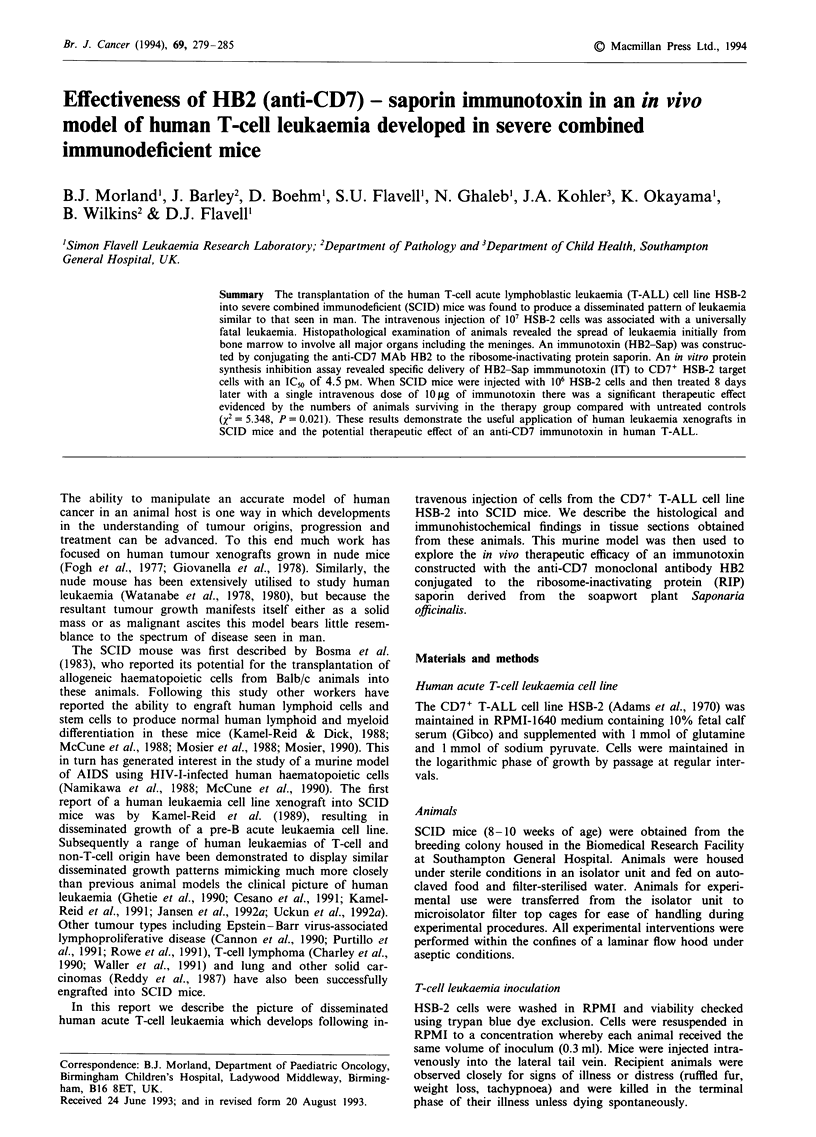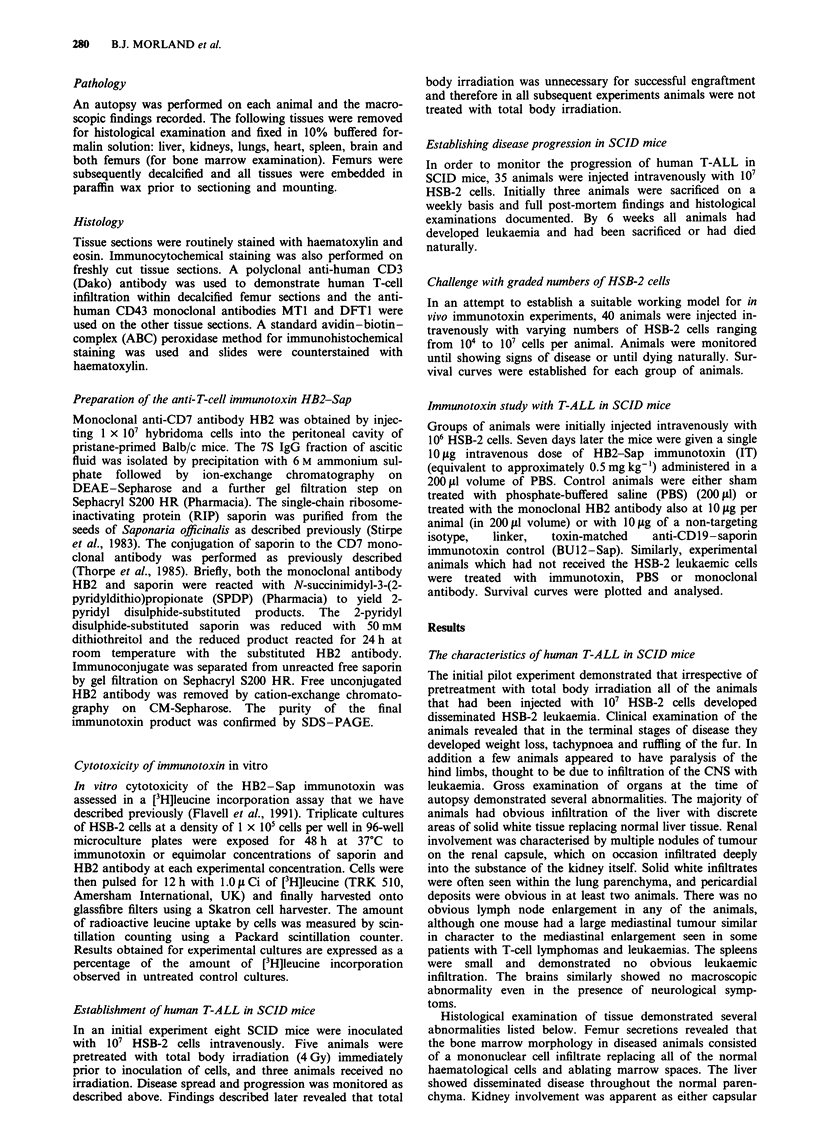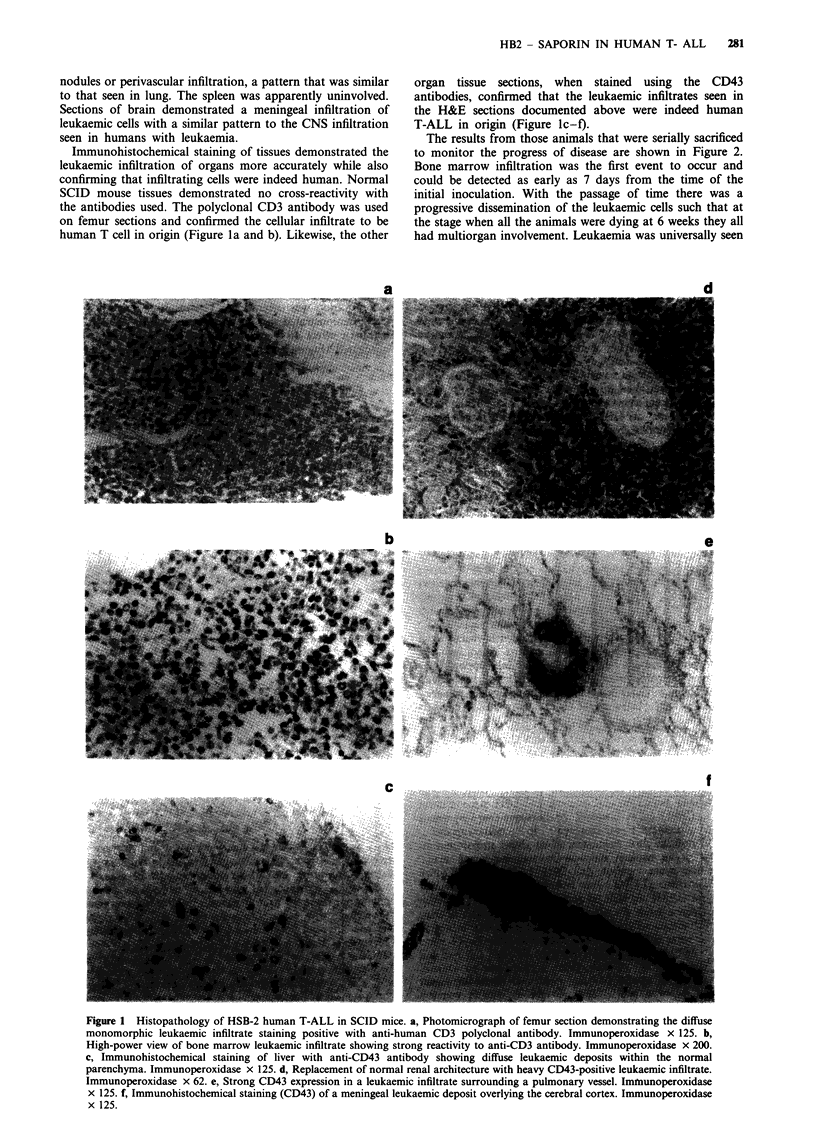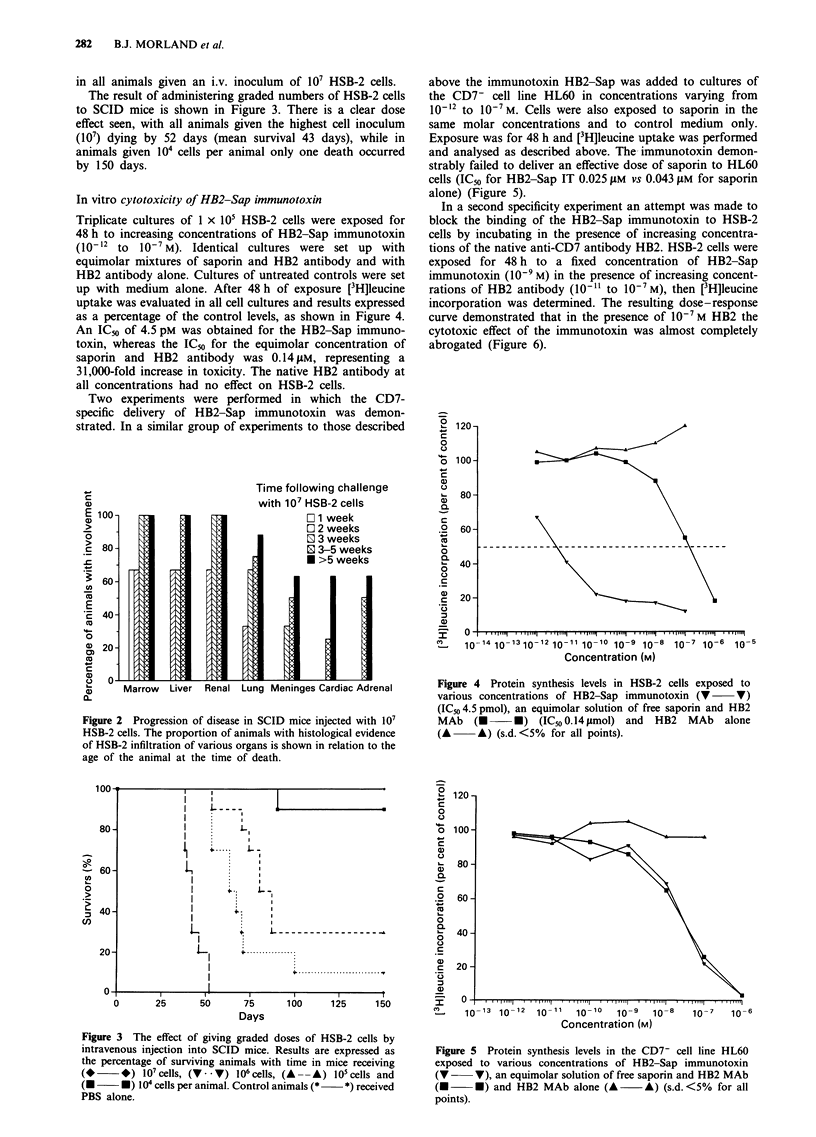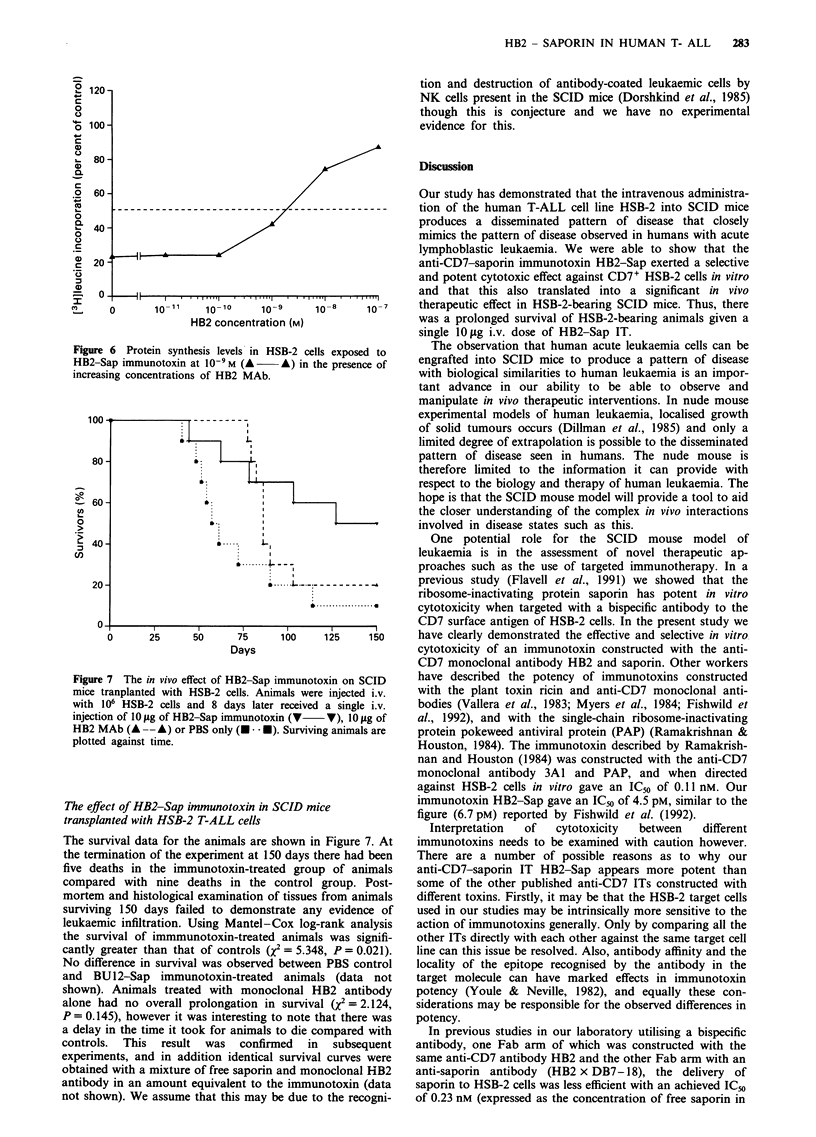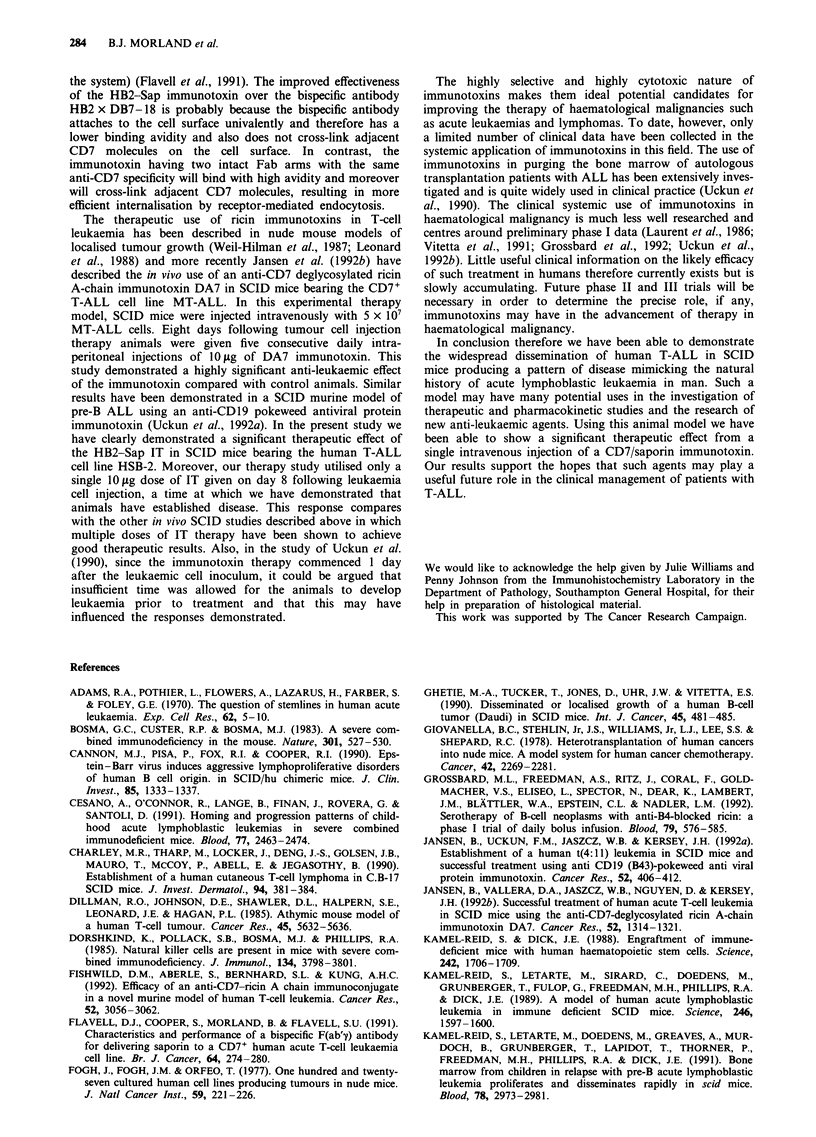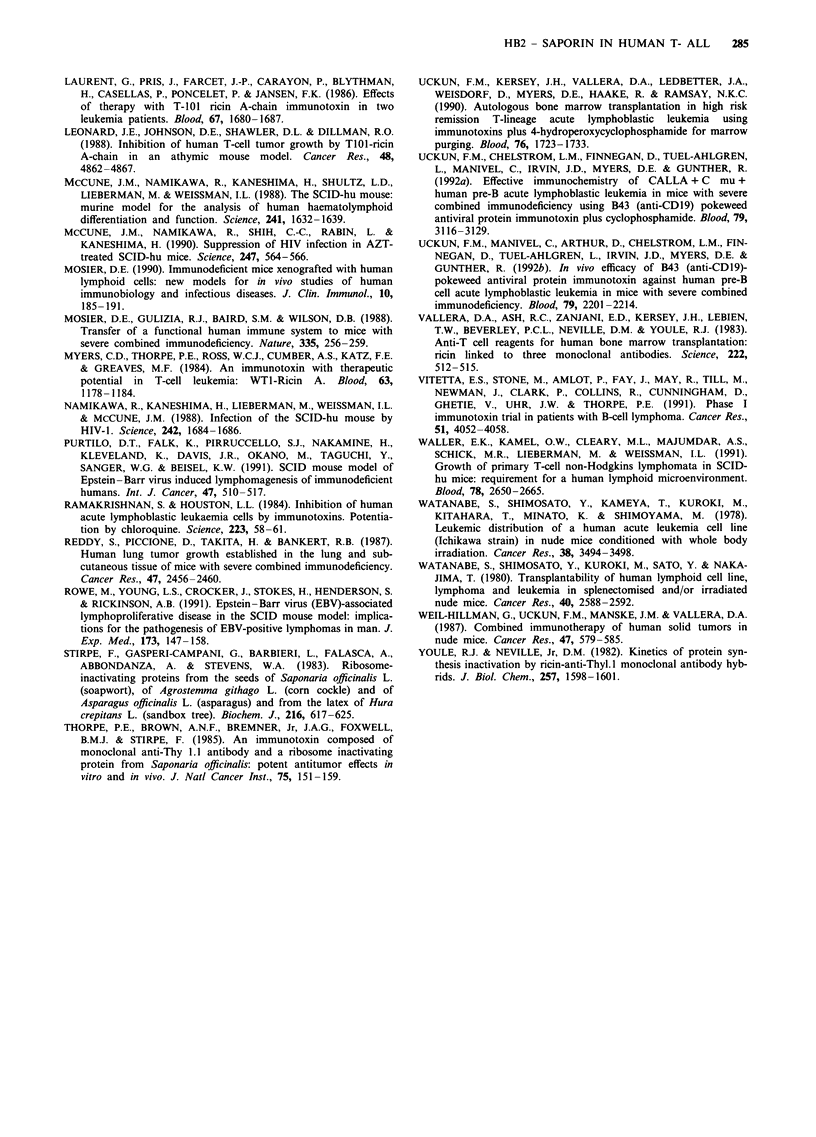# Effectiveness of HB2 (anti-CD7)--saporin immunotoxin in an in vivo model of human T-cell leukaemia developed in severe combined immunodeficient mice.

**DOI:** 10.1038/bjc.1994.52

**Published:** 1994-02

**Authors:** B. J. Morland, J. Barley, D. Boehm, S. U. Flavell, N. Ghaleb, J. A. Kohler, K. Okayama, B. Wilkins, D. J. Flavell

**Affiliations:** Simon Flavell Leukaemia Research Laboratory, Southampton General Hospital, UK.

## Abstract

**Images:**


Br. J. Cancer (1994), 69, 279-285

© Macmillan Press Ltd., 1994

### Effectiveness of HB2 (anti-CD7) – saporin immunotoxin in an *in vivo* model of human T-cell leukaemia developed in severe combined immunodeficient mice

B.J. Morland[1], J. Barley[2], D. Boehm[1], S.U. Flavell[1], N. Ghaleb[1], J.A. Kohler[3], K. Okayama[1], B. Wilkins[2] & D.J. Flavell[1]

[1]*Simon Flavell Leukaemia Research Laboratory;* [2]*Department of Pathology and* [3]*Department of Child Health, Southampton General Hospital, UK.*

**Summary** The transplantation of the human T-cell acute lymphoblastic leukaemia (T-ALL) cell line HSB-2 into severe combined immunodeficient (SCID) mice was found to produce a disseminated pattern of leukaemia similar to that seen in man. The intravenous injection of $10^7$ HSB-2 cells was associated with a universally fatal leukaemia. Histopathological examination of animals revealed the spread of leukaemia initially from bone marrow to involve all major organs including the meninges. An immunotoxin (HB2–Sap) was constructed by conjugating the anti-CD7 MAb HB2 to the ribosome-inactivating protein saporin. An *in vitro* protein synthesis inhibition assay revealed specific delivery of HB2–Sap immmunotoxin (IT) to $CD7^+$ HSB-2 target cells with an $IC_{50}$ of 4.5 pM. When SCID mice were injected with $10^6$ HSB-2 cells and then treated 8 days later with a single intravenous dose of 10 µg of immunotoxin there was a significant therapeutic effect evidenced by the numbers of animals surviving in the therapy group compared with untreated controls ($\chi^2 = 5.348$, $P = 0.021$). These results demonstrate the useful application of human leukaemia xenografts in SCID mice and the potential therapeutic effect of an anti-CD7 immunotoxin in human T-ALL.

The ability to manipulate an accurate model of human cancer in an animal host is one way in which developments in the understanding of tumour origins, progression and treatment can be advanced. To this end much work has focused on human tumour xenografts grown in nude mice (Fogh *et al.*, 1977; Giovanella *et al.*, 1978). Similarly, the nude mouse has been extensively utilised to study human leukaemia (Watanabe *et al.*, 1978, 1980), but because the resultant tumour growth manifests itself either as a solid mass or as malignant ascites this model bears little resemblance to the spectrum of disease seen in man.

The SCID mouse was first described by Bosma *et al.* (1983), who reported its potential for the transplantation of allogeneic haematopoietic cells from Balb/c animals into these animals. Following this study other workers have reported the ability to engraft human lymphoid cells and stem cells to produce normal human lymphoid and myeloid differentiation in these mice (Kamel-Reid & Dick, 1988; McCune *et al.*, 1988; Mosier *et al.*, 1988; Mosier, 1990). This in turn has generated interest in the study of a murine model of AIDS using HIV-I-infected human haematopoietic cells (Namikawa *et al.*, 1988; McCune *et al.*, 1990). The first report of a human leukaemia cell line xenograft into SCID mice was by Kamel-Reid *et al.* (1989), resulting in disseminated growth of a pre-B acute leukaemia cell line. Subsequently a range of human leukaemias of T-cell and non-T-cell origin have been demonstrated to display similar disseminated growth patterns mimicking much more closely than previous animal models the clinical picture of human leukaemia (Ghetie *et al.*, 1990; Cesano *et al.*, 1991; Kamel-Reid *et al.*, 1991; Jansen *et al.*, 1992*a*; Uckun *et al.*, 1992*a*). Other tumour types including Epstein–Barr virus-associated lymphoproliferative disease (Cannon *et al.*, 1990; Purtillo *et al.*, 1991; Rowe *et al.*, 1991), T-cell lymphoma (Charley *et al.*, 1990; Waller *et al.*, 1991) and lung and other solid carcinomas (Reddy *et al.*, 1987) have also been successfully engrafted into SCID mice.

In this report we describe the picture of disseminated human acute T-cell leukaemia which develops following intravenous injection of cells from the $CD7^+$ T-ALL cell line HSB-2 into SCID mice. We describe the histological and immunohistochemical findings in tissue sections obtained from these animals. This murine model was then used to explore the *in vivo* therapeutic efficacy of an immunotoxin constructed with the anti-CD7 monoclonal antibody HB2 conjugated to the ribosome-inactivating protein (RIP) saporin derived from the soapwort plant *Saponaria officinalis*.

Correspondence: B.J. Morland, Department of Paediatric Oncology, Birmingham Children's Hospital, Ladywood Middleway, Birmingham, B16 8ET, UK.
Received 24 June 1993; and in revised form 20 August 1993.

### Materials and methods

### *Human acute T-cell leukaemia cell line*

The $CD7^+$ T-ALL cell line HSB-2 (Adams *et al.*, 1970) was maintained in RPMI-1640 medium containing 10% fetal calf serum (Gibco) and supplemented with 1 mmol of glutamine and 1 mmol of sodium pyruvate. Cells were maintained in the logarithmic phase of growth by passage at regular intervals.

### *Animals*

SCID mice (8–10 weeks of age) were obtained from the breeding colony housed in the Biomedical Research Facility at Southampton General Hospital. Animals were housed under sterile conditions in an isolator unit and fed on autoclaved food and filter-sterilised water. Animals for experimental use were transferred from the isolator unit to microisolator filter top cages for ease of handling during experimental procedures. All experimental interventions were performed within the confines of a laminar flow hood under aseptic conditions.

### *T-cell leukaemia inoculation*

HSB-2 cells were washed in RPMI and viability checked using trypan blue dye exclusion. Cells were resuspended in RPMI to a concentration whereby each animal received the same volume of inoculum (0.3 ml). Mice were injected intravenously into the lateral tail vein. Recipient animals were observed closely for signs of illness or distress (ruffled fur, weight loss, tachypnoea) and were killed in the terminal phase of their illness unless dying spontaneously.

*Pathology*

An autopsy was performed on each animal and the macroscopic findings recorded. The following tissues were removed for histological examination and fixed in 10% buffered formalin solution: liver, kidneys, lungs, heart, spleen, brain and both femurs (for bone marrow examination). Femurs were subsequently decalcified and all tissues were embedded in paraffin wax prior to sectioning and mounting.

*Histology*

Tissue sections were routinely stained with haematoxylin and eosin. Immunocytochemical staining was also performed on freshly cut tissue sections. A polyclonal anti-human CD3 (Dako) antibody was used to demonstrate human T-cell infiltration within decalcified femur sections and the anti-human CD43 monoclonal antibodies MT1 and DFT1 were used on the other tissue sections. A standard avidin–biotin–complex (ABC) peroxidase method for immunohistochemical staining was used and slides were counterstained with haematoxylin.

*Preparation of the anti-T-cell immunotoxin HB2–Sap*

Monoclonal anti-CD7 antibody HB2 was obtained by injecting $1 \times 10^7$ hybridoma cells into the peritoneal cavity of pristane-primed Balb/c mice. The 7S IgG fraction of ascitic fluid was isolated by precipitation with 6 M ammonium sulphate followed by ion-exchange chromatography on DEAE–Sepharose and a further gel filtration step on Sephacryl S200 HR (Pharmacia). The single-chain ribosome-inactivating protein (RIP) saporin was purified from the seeds of *Saponaria officinalis* as described previously (Stirpe *et al.*, 1983). The conjugation of saporin to the CD7 monoclonal antibody was performed as previously described (Thorpe *et al.*, 1985). Briefly, both the monoclonal antibody HB2 and saporin were reacted with *N*-succinimidyl-3-(2-pyridyldithio)propionate (SPDP) (Pharmacia) to yield 2-pyridyl disulphide-substituted products. The 2-pyridyl disulphide-substituted saporin was reduced with 50 mM dithiothreitol and the reduced product reacted for 24 h at room temperature with the substituted HB2 antibody. Immunoconjugate was separated from unreacted free saporin by gel filtration on Sephacryl S200 HR. Free unconjugated HB2 antibody was removed by cation-exchange chromatography on CM-Sepharose. The purity of the final immunotoxin product was confirmed by SDS–PAGE.

*Cytotoxicity of immunotoxin* in vitro

*In vitro* cytotoxicity of the HB2–Sap immunotoxin was assessed in a [$^3$H]leucine incorporation assay that we have described previously (Flavell *et al.*, 1991). Triplicate cultures of HSB-2 cells at a density of $1 \times 10^5$ cells per well in 96-well microculture plates were exposed for 48 h at 37°C to immunotoxin or equimolar concentrations of saporin and HB2 antibody at each experimental concentration. Cells were then pulsed for 12 h with 1.0 $\mu$Ci of [$^3$H]leucine (TRK 510, Amersham International, UK) and finally harvested onto glassfibre filters using a Skatron cell harvester. The amount of radioactive leucine uptake by cells was measured by scintillation counting using a Packard scintillation counter. Results obtained for experimental cultures are expressed as a percentage of the amount of [$^3$H]leucine incorporation observed in untreated control cultures.

*Establishment of human T-ALL in SCID mice*

In an initial experiment eight SCID mice were inoculated with $10^7$ HSB-2 cells intravenously. Five animals were pretreated with total body irradiation (4 Gy) immediately prior to inoculation of cells, and three animals received no irradiation. Disease spread and progression was monitored as described above. Findings described later revealed that total body irradiation was unnecessary for successful engraftment and therefore in all subsequent experiments animals were not treated with total body irradiation.

*Establishing disease progression in SCID mice*

In order to monitor the progression of human T-ALL in SCID mice, 35 animals were injected intravenously with $10^7$ HSB-2 cells. Initially three animals were sacrificed on a weekly basis and full post-mortem findings and histological examinations documented. By 6 weeks all animals had developed leukaemia and had been sacrificed or had died naturally.

*Challenge with graded numbers of HSB-2 cells*

In an attempt to establish a suitable working model for *in vivo* immunotoxin experiments, 40 animals were injected intravenously with varying numbers of HSB-2 cells ranging from $10^4$ to $10^7$ cells per animal. Animals were monitored until showing signs of disease or until dying naturally. Survival curves were established for each group of animals.

*Immunotoxin study with T-ALL in SCID mice*

Groups of animals were initially injected intravenously with $10^6$ HSB-2 cells. Seven days later the mice were given a single 10 $\mu$g intravenous dose of HB2–Sap immunotoxin (IT) (equivalent to approximately 0.5 mg kg$^{-1}$) administered in a 200 $\mu$l volume of PBS. Control animals were either sham treated with phosphate-buffered saline (PBS) (200 $\mu$l) or treated with the monoclonal HB2 antibody also at 10 $\mu$g per animal (in 200 $\mu$l volume) or with 10 $\mu$g of a non-targeting isotype, linker, toxin-matched anti-CD19–saporin immunotoxin control (BU12–Sap). Similarly, experimental animals which had not received the HSB-2 leukaemic cells were treated with immunotoxin, PBS or monoclonal antibody. Survival curves were plotted and analysed.

### Results

*The characteristics of human T-ALL in SCID mice*

The initial pilot experiment demonstrated that irrespective of pretreatment with total body irradiation all of the animals that had been injected with $10^7$ HSB-2 cells developed disseminated HSB-2 leukaemia. Clinical examination of the animals revealed that in the terminal stages of disease they developed weight loss, tachypnoea and ruffling of the fur. In addition a few animals appeared to have paralysis of the hind limbs, thought to be due to infiltration of the CNS with leukaemia. Gross examination of organs at the time of autopsy demonstrated several abnormalities. The majority of animals had obvious infiltration of the liver with discrete areas of solid white tissue replacing normal liver tissue. Renal involvement was characterised by multiple nodules of tumour on the renal capsule, which on occasion infiltrated deeply into the substance of the kidney itself. Solid white infiltrates were often seen within the lung parenchyma, and pericardial deposits were obvious in at least two animals. There was no obvious lymph node enlargement in any of the animals, although one mouse had a large mediastinal tumour similar in character to the mediastinal enlargement seen in some patients with T-cell lymphomas and leukaemias. The spleens were small and demonstrated no obvious leukaemic infiltration. The brains similarly showed no macroscopic abnormality even in the presence of neurological symptoms.

Histological examination of tissue demonstrated several abnormalities listed below. Femur secretions revealed that the bone marrow morphology in diseased animals consisted of a mononuclear cell infiltrate replacing all of the normal haematological cells and ablating marrow spaces. The liver showed disseminated disease throughout the normal parenchyma. Kidney involvement was apparent as either capsular nodules or perivascular infiltration, a pattern that was similar to that seen in lung. The spleen was apparently uninvolved. Sections of brain demonstrated a meningeal infiltration of leukaemic cells with a similar pattern to the CNS infiltration seen in humans with leukaemia.

Immunohistochemical staining of tissues demonstrated the leukaemic infiltration of organs more accurately while also confirming that infiltrating cells were indeed human. Normal SCID mouse tissues demonstrated no cross-reactivity with the antibodies used. The polyclonal CD3 antibody was used on femur sections and confirmed the cellular infiltrate to be human T cell in origin (Figure 1a and b). Likewise, the other organ tissue sections, when stained using the CD43 antibodies, confirmed that the leukaemic infiltrates seen in the H&E sections documented above were indeed human T-ALL in origin (Figure 1c–f).

The results from those animals that were serially sacrificed to monitor the progress of disease are shown in Figure 2. Bone marrow infiltration was the first event to occur and could be detected as early as 7 days from the time of the initial inoculation. With the passage of time there was a progressive dissemination of the leukaemic cells such that at the stage when all the animals were dying at 6 weeks they all had multiorgan involvement. Leukaemia was universally seen

**Figure 1** Histopathology of HSB-2 human T-ALL in SCID mice. **a**, Photomicrograph of femur section demonstrating the diffuse monomorphic leukaemic infiltrate staining positive with anti-human CD3 polyclonal antibody. Immunoperoxidase × 125. **b**, High-power view of bone marrow leukaemic infiltrate showing strong reactivity to anti-CD3 antibody. Immunoperoxidase × 200. **c**, Immunohistochemical staining of liver with anti-CD43 antibody showing diffuse leukaemic deposits within the normal parenchyma. Immunoperoxidase × 125. **d**, Replacement of normal renal architecture with heavy CD43-positive leukaemic infiltrate. Immunoperoxidase × 62. **e**, Strong CD43 expression in a leukaemic infiltrate surrounding a pulmonary vessel. Immunoperoxidase × 125. **f**, Immunohistochemical staining (CD43) of a meningeal leukaemic deposit overlying the cerebral cortex. Immunoperoxidase × 125.

in all animals given an i.v. inoculum of $10^7$ HSB-2 cells.

The result of administering graded numbers of HSB-2 cells to SCID mice is shown in Figure 3. There is a clear dose effect seen, with all animals given the highest cell inoculum ($10^7$) dying by 52 days (mean survival 43 days), while in animals given $10^4$ cells per animal only one death occurred by 150 days.

### In vitro *cytotoxicity of HB2–Sap immunotoxin*

Triplicate cultures of $1 \times 10^5$ HSB-2 cells were exposed for 48 h to increasing concentrations of HB2–Sap immunotoxin ($10^{-12}$ to $10^{-7}$ M). Identical cultures were set up with equimolar mixtures of saporin and HB2 antibody and with HB2 antibody alone. Cultures of untreated controls were set up with medium alone. After 48 h of exposure [$^3$H]leucine uptake was evaluated in all cell cultures and results expressed as a percentage of the control levels, as shown in Figure 4. An $IC_{50}$ of 4.5 pM was obtained for the HB2–Sap immunotoxin, whereas the $IC_{50}$ for the equimolar concentration of saporin and HB2 antibody was 0.14 μM, representing a 31,000-fold increase in toxicity. The native HB2 antibody at all concentrations had no effect on HSB-2 cells.

Two experiments were performed in which the CD7-specific delivery of HB2–Sap immunotoxin was demonstrated. In a similar group of experiments to those described above the immunotoxin HB2–Sap was added to cultures of the CD7$^-$ cell line HL60 in concentrations varying from $10^{-12}$ to $10^{-7}$ M. Cells were also exposed to saporin in the same molar concentrations and to control medium only. Exposure was for 48 h and [$^3$H]leucine uptake was performed and analysed as described above. The immunotoxin demonstrably failed to deliver an effective dose of saporin to HL60 cells ($IC_{50}$ for HB2–Sap IT 0.025 μM *vs* 0.043 μM for saporin alone) (Figure 5).

In a second specificity experiment an attempt was made to block the binding of the HB2–Sap immunotoxin to HSB-2 cells by incubating in the presence of increasing concentrations of the native anti-CD7 antibody HB2. HSB-2 cells were exposed for 48 h to a fixed concentration of HB2–Sap immunotoxin ($10^{-9}$ M) in the presence of increasing concentrations of HB2 antibody ($10^{-11}$ to $10^{-7}$ M), then [$^3$H]leucine incorporation was determined. The resulting dose–response curve demonstrated that in the presence of $10^{-7}$ M HB2 the cytotoxic effect of the immunotoxin was almost completely abrogated (Figure 6).

**Figure 2** Progression of disease in SCID mice injected with $10^7$ HSB-2 cells. The proportion of animals with histological evidence of HSB-2 infiltration of various organs is shown in relation to the age of the animal at the time of death.

**Figure 3** The effect of giving graded doses of HSB-2 cells by intravenous injection into SCID mice. Results are expressed as the percentage of surviving animals with time in mice receiving (◆——◆) $10^7$ cells, (▼··▼) $10^6$ cells, (▲--▲) $10^5$ cells and (■——■) $10^4$ cells per animal. Control animals (*——*) received PBS alone.

**Figure 4** Protein synthesis levels in HSB-2 cells exposed to various concentrations of HB2–Sap immunotoxin (▼——▼) ($IC_{50}$ 4.5 pmol), an equimolar solution of free saporin and HB2 MAb (■——■) ($IC_{50}$ 0.14 μmol) and HB2 MAb alone (▲——▲) (s.d. <5% for all points).

**Figure 5** Protein synthesis levels in the CD7$^-$ cell line HL60 exposed to various concentrations of HB2–Sap immunotoxin (▼——▼), an equimolar solution of free saporin and HB2 MAb (■——■) and HB2 MAb alone (▲——▲) (s.d. <5% for all points).

**Figure 6** Protein synthesis levels in HSB-2 cells exposed to HB2–Sap immunotoxin at $10^{-9}$ M (▲——▲) in the presence of increasing concentrations of HB2 MAb.

**Figure 7** The *in vivo* effect of HB2–Sap immunotoxin on SCID mice tranplanted with HSB-2 cells. Animals were injected i.v. with $10^6$ HSB-2 cells and 8 days later received a single i.v. injection of 10 μg of HB2–Sap immunotoxin (▼——▼), 10 μg of HB2 MAb (▲ — ▲) or PBS only (■ · · ■). Surviving animals are plotted against time.

### *The effect of HB2–Sap immunotoxin in SCID mice transplanted with HSB-2 T-ALL cells*

The survival data for the animals are shown in Figure 7. At the termination of the experiment at 150 days there had been five deaths in the immunotoxin-treated group of animals compared with nine deaths in the control group. Post-mortem and histological examination of tissues from animals surviving 150 days failed to demonstrate any evidence of leukaemic infiltration. Using Mantel–Cox log-rank analysis the survival of immmunotoxin-treated animals was significantly greater than that of controls ($\chi^2 = 5.348$, $P = 0.021$). No difference in survival was observed between PBS control and BU12–Sap immunotoxin-treated animals (data not shown). Animals treated with monoclonal HB2 antibody alone had no overall prolongation in survival ($\chi^2 = 2.124$, $P = 0.145$), however it was interesting to note that there was a delay in the time it took for animals to die compared with controls. This result was confirmed in subsequent experiments, and in addition identical survival curves were obtained with a mixture of free saporin and monoclonal HB2 antibody in an amount equivalent to the immunotoxin (data not shown). We assume that this may be due to the recognition and destruction of antibody-coated leukaemic cells by NK cells present in the SCID mice (Dorshkind *et al.*, 1985) though this is conjecture and we have no experimental evidence for this.

### Discussion

Our study has demonstrated that the intravenous administration of the human T-ALL cell line HSB-2 into SCID mice produces a disseminated pattern of disease that closely mimics the pattern of disease observed in humans with acute lymphoblastic leukaemia. We were able to show that the anti-CD7–saporin immunotoxin HB2–Sap exerted a selective and potent cytotoxic effect against $CD7^+$ HSB-2 cells *in vitro* and that this also translated into a significant *in vivo* therapeutic effect in HSB-2-bearing SCID mice. Thus, there was a prolonged survival of HSB-2-bearing animals given a single 10 μg i.v. dose of HB2–Sap IT.

The observation that human acute leukaemia cells can be engrafted into SCID mice to produce a pattern of disease with biological similarities to human leukaemia is an important advance in our ability to be able to observe and manipulate *in vivo* therapeutic interventions. In nude mouse experimental models of human leukaemia, localised growth of solid tumours occurs (Dillman *et al.*, 1985) and only a limited degree of extrapolation is possible to the disseminated pattern of disease seen in humans. The nude mouse is therefore limited to the information it can provide with respect to the biology and therapy of human leukaemia. The hope is that the SCID mouse model will provide a tool to aid the closer understanding of the complex *in vivo* interactions involved in disease states such as this.

One potential role for the SCID mouse model of leukaemia is in the assessment of novel therapeutic approaches such as the use of targeted immunotherapy. In a previous study (Flavell *et al.*, 1991) we showed that the ribosome-inactivating protein saporin has potent *in vitro* cytotoxicity when targeted with a bispecific antibody to the CD7 surface antigen of HSB-2 cells. In the present study we have clearly demonstrated the effective and selective *in vitro* cytotoxicity of an immunotoxin constructed with the anti-CD7 monoclonal antibody HB2 and saporin. Other workers have described the potency of immunotoxins constructed with the plant toxin ricin and anti-CD7 monoclonal antibodies (Vallera *et al.*, 1983; Myers *et al.*, 1984; Fishwild *et al.*, 1992), and with the single-chain ribosome-inactivating protein pokeweed antiviral protein (PAP) (Ramakrishnan & Houston, 1984). The immunotoxin described by Ramakrishnan and Houston (1984) was constructed with the anti-CD7 monoclonal antibody 3A1 and PAP, and when directed against HSB-2 cells *in vitro* gave an $IC_{50}$ of 0.11 nM. Our immunotoxin HB2–Sap gave an $IC_{50}$ of 4.5 pM, similar to the figure (6.7 pM) reported by Fishwild *et al.* (1992).

Interpretation of cytotoxicity between different immunotoxins needs to be examined with caution however. There are a number of possible reasons as to why our anti-CD7–saporin IT HB2–Sap appears more potent than some of the other published anti-CD7 ITs constructed with different toxins. Firstly, it may be that the HSB-2 target cells used in our studies may be intrinsically more sensitive to the action of immunotoxins generally. Only by comparing all the other ITs directly with each other against the same target cell line can this issue be resolved. Also, antibody affinity and the locality of the epitope recognised by the antibody in the target molecule can have marked effects in immunotoxin potency (Youle & Neville, 1982), and equally these considerations may be responsible for the observed differences in potency.

In previous studies in our laboratory utilising a bispecific antibody, one Fab arm of which was constructed with the same anti-CD7 antibody HB2 and the other Fab arm with an anti-saporin antibody (HB2 × DB7–18), the delivery of saporin to HSB-2 cells was less efficient with an achieved $IC_{50}$ of 0.23 nM (expressed as the concentration of free saporin in the system) (Flavell et al., 1991). The improved effectiveness of the HB2–Sap immunotoxin over the bispecific antibody HB2 × DB7–18 is probably because the bispecific antibody attaches to the cell surface univalently and therefore has a lower binding avidity and also does not cross-link adjacent CD7 molecules on the cell surface. In contrast, the immunotoxin having two intact Fab arms with the same anti-CD7 specificity will bind with high avidity and moreover will cross-link adjacent CD7 molecules, resulting in more efficient internalisation by receptor-mediated endocytosis.

The therapeutic use of ricin immunotoxins in T-cell leukaemia has been described in nude mouse models of localised tumour growth (Weil-Hilman et al., 1987; Leonard et al., 1988) and more recently Jansen et al. (1992b) have described the *in vivo* use of an anti-CD7 deglycosylated ricin A-chain immunotoxin DA7 in SCID mice bearing the $CD7^+$ T-ALL cell line MT-ALL. In this experimental therapy model, SCID mice were injected intravenously with $5 \times 10^7$ MT-ALL cells. Eight days following tumour cell injection therapy animals were given five consecutive daily intraperitoneal injections of 10 µg of DA7 immunotoxin. This study demonstrated a highly significant anti-leukaemic effect of the immunotoxin compared with control animals. Similar results have been demonstrated in a SCID murine model of pre-B ALL using an anti-CD19 pokeweed antiviral protein immunotoxin (Uckun et al., 1992a). In the present study we have clearly demonstrated a significant therapeutic effect of the HB2–Sap IT in SCID mice bearing the human T-ALL cell line HSB-2. Moreover, our therapy study utilised only a single 10 µg dose of IT given on day 8 following leukaemia cell injection, a time at which we have demonstrated that animals have established disease. This response compares with the other *in vivo* SCID studies described above in which multiple doses of IT therapy have been shown to achieve good therapeutic results. Also, in the study of Uckun et al. (1990), since the immunotoxin therapy commenced 1 day after the leukaemic cell inoculum, it could be argued that insufficient time was allowed for the animals to develop leukaemia prior to treatment and that this may have influenced the responses demonstrated.

The highly selective and highly cytotoxic nature of immunotoxins makes them ideal potential candidates for improving the therapy of haematological malignancies such as acute leukaemias and lymphomas. To date, however, only a limited number of clinical data have been collected in the systemic application of immunotoxins in this field. The use of immunotoxins in purging the bone marrow of autologous transplantation patients with ALL has been extensively investigated and is quite widely used in clinical practice (Uckun et al., 1990). The clinical systemic use of immunotoxins in haematological malignancy is much less well researched and centres around preliminary phase I data (Laurent et al., 1986; Vitetta et al., 1991; Grossbard et al., 1992; Uckun et al., 1992b). Little useful clinical information on the likely efficacy of such treatment in humans therefore currently exists but is slowly accumulating. Future phase II and III trials will be necessary in order to determine the precise role, if any, immunotoxins may have in the advancement of therapy in haematological malignancy.

In conclusion therefore we have been able to demonstrate the widespread dissemination of human T-ALL in SCID mice producing a pattern of disease mimicking the natural history of acute lymphoblastic leukaemia in man. Such a model may have many potential uses in the investigation of therapeutic and pharmacokinetic studies and the research of new anti-leukaemic agents. Using this animal model we have been able to show a significant therapeutic effect from a single intravenous injection of a CD7/saporin immunotoxin. Our results support the hopes that such agents may play a useful future role in the clinical management of patients with T-ALL.

We would like to acknowledge the help given by Julie Williams and Penny Johnson from the Immunohistochemistry Laboratory in the Department of Pathology, Southampton General Hospital, for their help in preparation of histological material.

This work was supported by The Cancer Research Campaign.

### References

ADAMS, R.A., POTHIER, L., FLOWERS, A., LAZARUS, H., FARBER, S. & FOLEY, G.E. (1970). The question of stemlines in human acute leukaemia. *Exp. Cell Res.*, **62**, 5–10.

BOSMA, G.C., CUSTER, R.P. & BOSMA, M.J. (1983). A severe combined immunodeficiency in the mouse. *Nature*, **301**, 527–530.

CANNON, M.J., PISA, P., FOX, R.I. & COOPER, R.I. (1990). Epstein–Barr virus induces aggressive lymphoproliferative disorders of human B cell origin. in SCID/hu chimeric mice. *J. Clin. Invest.*, **85**, 1333–1337.

CESANO, A., O'CONNOR, R., LANGE, B., FINAN, J., ROVERA, G. & SANTOLI, D. (1991). Homing and progression patterns of childhood acute lymphoblastic leukemias in severe combined immunodeficient mice. *Blood*, **77**, 2463–2474.

CHARLEY, M.R., THARP, M., LOCKER, J., DENG, J.-S., GOLSEN, J.B., MAURO, T., MCCOY, P., ABELL, E. & JEGASOTHY, B. (1990). Establishment of a human cutaneous T-cell lymphoma in C.B-17 SCID mice. *J. Invest. Dermatol.*, **94**, 381–384.

DILLMAN, R.O., JOHNSON, D.E., SHAWLER, D.L., HALPERN, S.E., LEONARD, J.E. & HAGAN, P.L. (1985). Athymic mouse model of a human T-cell tumour. *Cancer Res.*, **45**, 5632–5636.

DORSHKIND, K., POLLACK, S.B., BOSMA, M.J. & PHILLIPS, R.A. (1985). Natural killer cells are present in mice with severe combined immunodeficiency. *J. Immunol.*, **134**, 3798–3801.

FISHWILD, D.M., ABERLE, S., BERNHARD, S.L. & KUNG, A.H.C. (1992). Efficacy of an anti-CD7–ricin A chain immunoconjugate in a novel murine model of human T-cell leukemia. *Cancer Res.*, **52**, 3056–3062.

FLAVELL, D.J., COOPER, S., MORLAND, B. & FLAVELL, S.U. (1991). Characteristics and performance of a bispecific F(ab'γ) antibody for delivering saporin to a $CD7^+$ human acute T-cell leukaemia cell line. *Br. J. Cancer*, **64**, 274–280.

FOGH, J., FOGH, J.M. & ORFEO, T. (1977). One hundred and twenty-seven cultured human cell lines producing tumours in nude mice. *J. Natl Cancer Inst.*, **59**, 221–226.

GHETIE, M.-A., TUCKER, T., JONES, D., UHR, J.W. & VITETTA, E.S. (1990). Disseminated or localised growth of a human B-cell tumor (Daudi) in SCID mice. *Int. J. Cancer*, **45**, 481–485.

GIOVANELLA, B.C., STEHLIN, Jr, J.S., WILLIAMS, Jr, L.J., LEE, S.S. & SHEPARD, R.C. (1978). Heterotransplantation of human cancers into nude mice. A model system for human cancer chemotherapy. *Cancer*, **42**, 2269–2281.

GROSSBARD, M.L., FREEDMAN, A.S., RITZ, J., CORAL, F., GOLDMACHER, V.S., ELISEO, L., SPECTOR, N., DEAR, K., LAMBERT, J.M., BLÄTTLER, W.A., EPSTEIN, C.L. & NADLER, L.M. (1992). Serotherapy of B-cell neoplasms with anti-B4-blocked ricin: a phase I trial of daily bolus infusion. *Blood*, **79**, 576–585.

JANSEN, B., UCKUN, F.M., JASZCZ, W.B. & KERSEY, J.H. (1992a). Establishment of a human t(4:11) leukemia in SCID mice and successful treatment using anti CD19 (B43)-pokeweed anti viral protein immunotoxin. *Cancer Res.*, **52**, 406–412.

JANSEN, B., VALLERA, D.A., JASZCZ, W.B., NGUYEN, D. & KERSEY, J.H. (1992b). Successful treatment of human acute T-cell leukemia in SCID mice using the anti-CD7-deglycosylated ricin A-chain immunotoxin DA7. *Cancer Res.*, **52**, 1314–1321.

KAMEL-REID, S. & DICK, J.E. (1988). Engraftment of immune-deficient mice with human haematopoietic stem cells. *Science*, **242**, 1706–1709.

KAMEL-REID, S., LETARTE, M., SIRARD, C., DOEDENS, M., GRUNBERGER, T., FULOP, G., FREEDMAN, M.H., PHILLIPS, R.A. & DICK, J.E. (1989). A model of human acute lymphoblastic leukemia in immune deficient SCID mice. *Science*, **246**, 1597–1600.

KAMEL-REID, S., LETARTE, M., DOEDENS, M., GREAVES, A., MURDOCH, B., GRUNBERGER, T., LAPIDOT, T., THORNER, P., FREEDMAN, M.H., PHILLIPS, R.A. & DICK, J.E. (1991). Bone marrow from children in relapse with pre-B acute lymphoblastic leukemia proliferates and disseminates rapidly in *scid* mice. *Blood*, **78**, 2973–2981.

LAURENT, G., PRIS, J., FARCET, J.-P., CARAYON, P., BLYTHMAN, H., CASELLAS, P., PONCELET, P. & JANSEN, F.K. (1986). Effects of therapy with T-101 ricin A-chain immunotoxin in two leukemia patients. *Blood*, **67**, 1680–1687.

LEONARD, J.E., JOHNSON, D.E., SHAWLER, D.L. & DILLMAN, R.O. (1988). Inhibition of human T-cell tumor growth by T101-ricin A-chain in an athymic mouse model. *Cancer Res.*, **48**, 4862–4867.

McCUNE, J.M., NAMIKAWA, R., KANESHIMA, H., SHULTZ, L.D., LIEBERMAN, M. & WEISSMAN, I.L. (1988). The SCID-hu mouse: murine model for the analysis of human haematolymphoid differentiation and function. *Science*, **241**, 1632–1639.

McCUNE, J.M., NAMIKAWA, R., SHIH, C.-C., RABIN, L. & KANESHIMA, H. (1990). Suppression of HIV infection in AZT-treated SCID-hu mice. *Science*, **247**, 564–566.

MOSIER, D.E. (1990). Immunodeficient mice xenografted with human lymphoid cells: new models for *in vivo* studies of human immunobiology and infectious diseases. *J. Clin. Immunol.*, **10**, 185–191.

MOSIER, D.E., GULIZIA, R.J., BAIRD, S.M. & WILSON, D.B. (1988). Transfer of a functional human immune system to mice with severe combined immunodeficiency. *Nature*, **335**, 256–259.

MYERS, C.D., THORPE, P.E., ROSS, W.C.J., CUMBER, A.S., KATZ, F.E. & GREAVES, M.F. (1984). An immunotoxin with therapeutic potential in T-cell leukemia: WT1-Ricin A. *Blood*, **63**, 1178–1184.

NAMIKAWA, R., KANESHIMA, H., LIEBERMAN, M., WEISSMAN, I.L. & McCUNE, J.M. (1988). Infection of the SCID-hu mouse by HIV-1. *Science*, **242**, 1684–1686.

PURTILO, D.T., FALK, K., PIRRUCCELLO, S.J., NAKAMINE, H., KLEVELAND, K., DAVIS, J.R., OKANO, M., TAGUCHI, Y., SANGER, W.G. & BEISEL, K.W. (1991). SCID mouse model of Epstein–Barr virus induced lymphomagenesis of immunodeficient humans. *Int. J. Cancer*, **47**, 510–517.

RAMAKRISHNAN, S. & HOUSTON, L.L. (1984). Inhibition of human acute lymphoblastic leukaemia cells by immunotoxins. Potentiation by chloroquine. *Science*, **223**, 58–61.

REDDY, S., PICCIONE, D., TAKITA, H. & BANKERT, R.B. (1987). Human lung tumor growth established in the lung and subcutaneous tissue of mice with severe combined immunodeficiency. *Cancer Res.*, **47**, 2456–2460.

ROWE, M., YOUNG, L.S., CROCKER, J., STOKES, H., HENDERSON, S. & RICKINSON, A.B. (1991). Epstein–Barr virus (EBV)-associated lymphoproliferative disease in the SCID mouse model: implications for the pathogenesis of EBV-positive lymphomas in man. *J. Exp. Med.*, **173**, 147–158.

STIRPE, F., GASPERI-CAMPANI, G., BARBIERI, L., FALASCA, A., ABBONDANZA, A. & STEVENS, W.A. (1983). Ribosome-inactivating proteins from the seeds of *Saponaria officinalis* L. (soapwort), of *Agrostemma githago* L. (corn cockle) and of *Asparagus officinalis* L. (asparagus) and from the latex of *Hura crepitans* L. (sandbox tree). *Biochem. J.*, **216**, 617–625.

THORPE, P.E., BROWN, A.N.F., BREMNER, Jr, J.A.G., FOXWELL, B.M.J. & STIRPE, F. (1985). An immunotoxin composed of monoclonal anti-Thy 1.1 antibody and a ribosome inactivating protein from *Saponaria officinalis*: potent antitumor effects *in vitro* and *in vivo*. *J. Natl Cancer Inst.*, **75**, 151–159.

UCKUN, F.M., KERSEY, J.H., VALLERA, D.A., LEDBETTER, J.A., WEISDORF, D., MYERS, D.E., HAAKE, R. & RAMSAY, N.K.C. (1990). Autologous bone marrow transplantation in high risk remission T-lineage acute lymphoblastic leukemia using immunotoxins plus 4-hydroperoxycyclophosphamide for marrow purging. *Blood*, **76**, 1723–1733.

UCKUN, F.M., CHELSTROM, L.M., FINNEGAN, D., TUEL-AHLGREN, L., MANIVEL, C., IRVIN, J.D., MYERS, D.E. & GUNTHER, R. (1992*a*). Effective immunochemistry of CALLA + C mu + human pre-B acute lymphoblastic leukemia in mice with severe combined immunodeficiency using B43 (anti-CD19) pokeweed antiviral protein immunotoxin plus cyclophosphamide. *Blood*, **79**, 3116–3129.

UCKUN, F.M., MANIVEL, C., ARTHUR, D., CHELSTROM, L.M., FINNEGAN, D., TUEL-AHLGREN, L., IRVIN, J.D., MYERS, D.E. & GUNTHER, R. (1992*b*). *In vivo* efficacy of B43 (anti-CD19)-pokeweed antiviral protein immunotoxin against human pre-B cell acute lymphoblastic leukemia in mice with severe combined immunodeficiency. *Blood*, **79**, 2201–2214.

VALLERA, D.A., ASH, R.C., ZANJANI, E.D., KERSEY, J.H., LEBIEN, T.W., BEVERLEY, P.C.L., NEVILLE, D.M. & YOULE, R.J. (1983). Anti-T cell reagents for human bone marrow transplantation: ricin linked to three monoclonal antibodies. *Science*, **222**, 512–515.

VITETTA, E.S., STONE, M., AMLOT, P., FAY, J., MAY, R., TILL, M., NEWMAN, J., CLARK, P., COLLINS, R., CUNNINGHAM, D., GHETIE, V., UHR, J.W. & THORPE, P.E. (1991). Phase I immunotoxin trial in patients with B-cell lymphoma. *Cancer Res.*, **51**, 4052–4058.

WALLER, E.K., KAMEL, O.W., CLEARY, M.L., MAJUMDAR, A.S., SCHICK, M.R., LIEBERMAN, M. & WEISSMAN, I.L. (1991). Growth of primary T-cell non-Hodgkins lymphomata in SCID-hu mice: requirement for a human lymphoid microenvironment. *Blood*, **78**, 2650–2665.

WATANABE, S., SHIMOSATO, Y., KAMEYA, T., KUROKI, M., KITAHARA, T., MINATO, K. & SHIMOYAMA, M. (1978). Leukemic distribution of a human acute leukemia cell line (Ichikawa strain) in nude mice conditioned with whole body irradiation. *Cancer Res.*, **38**, 3494–3498.

WATANABE, S., SHIMOSATO, Y., KUROKI, M., SATO, Y. & NAKAJIMA, T. (1980). Transplantability of human lymphoid cell line, lymphoma and leukemia in splenectomised and/or irradiated nude mice. *Cancer Res.*, **40**, 2588–2592.

WEIL-HILLMAN, G., UCKUN, F.M., MANSKE, J.M. & VALLERA, D.A. (1987). Combined immunotherapy of human solid tumors in nude mice. *Cancer Res.*, **47**, 579–585.

YOULE, R.J. & NEVILLE, Jr, D.M. (1982). Kinetics of protein synthesis inactivation by ricin-anti-Thyl.1 monoclonal antibody hybrids. *J. Biol. Chem.*, **257**, 1598–1601.